# Addressing Pressing Needs in the Development of Advanced Therapies

**DOI:** 10.3389/fbioe.2017.00055

**Published:** 2017-09-25

**Authors:** David Morrow, Anton Ussi, Giovanni Migliaccio

**Affiliations:** ^1^EATRIS ERIC, European Infrastructure for Translational Medicine, Amsterdam, Netherlands

**Keywords:** advanced therapy medicinal product, manufacturing technology, reimbursement mechanisms, funding models, regulatory pathway

## Abstract

The commercial development of advanced therapy medicinal products (ATMPs) represents great opportunity for therapeutic innovation but is beset by many challenges for its developers. Although the ATMP field continues to progress at a rapid pace, evidenced by the increasing number of clinical trials conducted over the past few years, several factors continue to complicate the introduction of ATMPs as a curative treatment for multiple disease types, by blocking their translational pathway from research to the patient. While several recent publications (Trounson and McDonald, 2015; Abou-El-Enein et al., 2016a,b) as well as an Innovative Medicines Initiative consultation (IMI, 2016) this year have highlighted the major gaps in ATMP development, with manufacturing, regulatory, and reimbursement issues at the forefront, there remains to be formulated a coherent strategy to address these by bringing the relevant stakeholders to a single forum, whose task it would be to design and execute a delta plan to alleviate the most pressing bottlenecks. This article focuses on two of the most urgent areas in need of attention in ATMP development, namely manufacturing and reimbursement, and promotes the concept of innovation-dedicated research infrastructures to support a multi-sector approach for ensuring the successful development, entry, and ensuing survival of ATMPs in the healthcare market.

## Hype and Reality—A Brief History of Advanced Therapy Medicinal Product (ATMP) Development

According to much of the popular media, the advent of stem cell-based therapies will increase therapeutic options in a range of indication areas, from cancer to fertility, fomenting a market revolution in the approach to treat almost all diseases. This hype is especially pervasive for degenerative and genetic diseases, which are the focus of the attention of rich societies (Kamenova and Caulfield, 2015).

The field created several new terms, such as “Stem Cell Therapies” and “Regenerative Medicine,” to label these new approaches. The hype started talking about immortality and replacing the aged parts of our bodies with cloned “young” organs and tissues created *in vitro*. Sadly, reality is a mere shadow of the hype.

In fact, many clinical trials were performed with encouraging clinical results (Heathman et al., 2015), but more often than not with the product poorly defined or with a lack of understanding of the mechanism of action. The abundance of intriguing cases of extremely successful treatments made the temptation to continue experimentation impossible to resist.

The name of ATMP was introduced by the European Commission Regulation 1394/2007, although medicinal products using either cells or genes were already in development for many years. The development of these products, however, was predominantly an academic affair (Thomson et al., 1998), based on the field’s advances in molecular editing and culture techniques. The regulatory environment was sharply refocused by the appearance of embryonic stem cells and their potential for manipulation *in vitro* (Thomson et al., 1998). The bioethical issues linked to their embryo origin and the *in vitro* fertilization techniques used for the creation of dedicated embryos (Wainwright et al., 2006) raised numerous concerns. In response to these rapid developments in the applied technologies, the European Parliament passed the Regulation 1394/2007 on the proviso that it would enter into force in 2008, and that a subsequent transitory period of three years would be granted for the registration of products already on the market at national level.

This estimation of the time required to reach compliance with the centralized Market Authorization process was hugely optimistic and, up to 2017, we still have only six licensed ATMPs, with three of them retired from the market for commercial reasons (PEI, 2016). In the USA, the FDA Office for Cellular, Tissue and Gene Therapies has granted licenses for seven products which are similar to the European ATMPs (FDA, 2017).

A critical development in the field, as usual, would come with new technology. The advent of novel immunotherapies, through the application of the chimeric antigen receptor T-cell gene editing technology was a game-changing event (Fellmann et al., 2017). These new products presented clear market potential by way of outstanding clinical results, which are easier to understand in the regulatory process and a clear mode of action. In fact, the CAR-T-cells can be seen as an evolution of the much-vaunted drug-conjugated antibodies, only with a “drug” that is a living cell. Nevertheless, the long-term viability of these new products needs to be assessed as the business model to be applied is untested with respect to manufacturing and reimbursement.

## Current Challenges in Advanced Therapy Development

The relative dearth of industrial investment in the ATMP space arguably lies behind the manifold bottlenecks in the ATMP space, especially more downstream aspects, such as manufacturing, control, distribution, and the various market-related challenges. The issues are numerous but here we will focus on distribution, manufacturing, and reimbursement.

### Distribution Models

How do we make a distributable ATMP? Firstly, there exists a real need to be able to decentralize ATMP manufacturing. Thus, it is imperative that the origin, composition, manufacturing process, QC methods, and batch release specifications of ATMPs must be aligned between regulatory agencies and ATMP manufacturers (Advanced Therapies Manufacturing Taskforce, 2016). Consistency of approach to utilization of GMP grade materials derived from well-characterized cell banks is also still a major issue, which is resulting in a failure to ensure consistency, safety, and purity of the final ATMPs. Moreover, additional complexities such as the shorter shelf life of cell therapies compared with other biologicals make these products particularly susceptible to damage during shipping, which contributes to the final quality of these products. One such example is Holoclar, where patient biopsies must be received by the manufacturer within 24 h following procurement and which has only a 36-h shelf life. In addition, Provenge has only an 18-h shelf life in a cool, insulated container and must be infused with three hours of opening (Abou-El-Enein et al., 2016b). Due to the temperamental nature of these products, one suggestion has been the establishment of regional sites or centers of excellence, which could offer a more suitable model for personalized autologous cell products or for rare diseases. Of course, consistency of approach and the necessary standards and guidelines would have to be conserved across these different regional sites and agreed on by the necessary regulatory authorities.

### Scaling-Up and Automating the Manufacturing Process

There are presently few realistic ideas for the cost-effective scale up of ATMPs (Heathman et al., 2015). Most of the development to date has been done in small volume vessels with manual processing. Such an approach, while feasible for small scale production, raises the overall cost of manufacturing and leads to quality and consistency issues, not least through the presence of human operators.

The very real need for “good enough” technology for driving reproducibility and standards that won’t break the bank for the developer is enhanced if the manufacturing process has to be duplicated in multiple site. With mesenchymal stem cells, for example, it is presently not commercially feasible to scale up sufficiently to be able to meet significant geographic spread of demand, also due to stringent requirements to show comparability of process across different manufacturing sites for a medicinal product. Hence a strategy to introduce methodology for quality confidence at the right time at an acceptable cost is fundamental, and any such efforts will need close coordination with the relevant regulatory instances. The development of automated, self-containing bioreactors is one of the possible approaches. However, very little has thus far been done to reliably demonstrate the bioequivalence of these bioreactor products with the manual procedure. Recent changes in the manufacturing guidelines have assisted in this critical gap in ATMP production but manufacturers have still failed to sufficiently address the critical requirements to meet increasing quality and safety concerns (Hourd et al., 2014; Papadaki, 2017).

Operations modeling is needed to investigate different modalities of production and distribution, such as production on-site in hospital networks or, for example, at fewer, more specialized regional centers of excellence. Establishment of registries at these sites and the maintenance of consolidated ATMP postmarketing follow-up databases should also be established. These modes of production would require new value chains with supply, manufacture, application, and storage of ATMP being established with these business models in mind from the very beginning of the development process. To establish new operational models, regulators must be engaged early on with the present drivers of ATMP development, namely academia, their spin-out ventures, and the relatively few industrial parties that have moved into the space.

### Reimbursement

Only part of the overall direct cost of manufacturing of these products is linked to the ultimate “sticker price” charged to payors. In fact, the cost of preclinical and clinical development before marketing authorization is quite a large fraction of the overall cost. The lack of accepted standards for efficacy and toxicity in the preclinical development phase is one issue driving these development costs. To ensure safety, the regulators should create a database of safety data related to manufacturing and testing which is non-existent now. Sometimes, as exemplified by ambiguity in the difference between senescence and precancerous stage, basic science also needs to be further advanced. Such gaps in understanding makes the development time uncertain and the process risky (Papadaki, 2017; Phacilitate, 2017).

Another issue is the necessity to provide data for each single product, depending on the type of manufacturing process used. The lack of a clear identity for cell-based preparations has forced the regulators to use the manufacturing process as a substitute (Sensebé et al., 2013). However, it is well known that small changes in the microenvironment result in modifications of gene expression and behavior in the cultured cells. In the absence of a clear understanding of what changes are relevant, all the changes in the manufacturing may alter the identity of the product, resulting in high cost for validation and uncertainty on the value of the intellectual property underlying the medicinal product.

In drug development, the pricing strategy will always be predominantly linked to the perceived market acceptance of a price, with factors such as competing products, market size, and patient benefit playing a major role. The complexity around the pricing of advanced therapies is largely due to the clinical effectiveness of one ATMP versus another not being known or quantifiable, as these therapies often target diseases which have limited to no alternative treatment options. As a result, there are no previous data for accurate health technology assessment (HTA) and appropriate pricing strategies, further complicating the process and limiting the options for a clear reimbursement strategy (Driscoll et al., 2017).

Can we assume therefore that the current HTA methodologies and frameworks in place cannot work for ATMPs? In comparison, current HTA methodologies to decide reimbursement for orphan medications differ widely, due to different strategies adopted by the healthcare payors in different countries. This includes use of cost effectiveness versus human value, for example, from one country to another. Upfront, one-time payments, especially for those treatments that may provide a permanent or very long-term “one-shot” curative effect, have been suggested but have appeared unpopular due to their perceived excessive nature. Glybera, an adeno-associated virus-mediated *in vivo* gene therapy that compensates lipoprotein lipase in patients with familial lipoprotein lipase deficiency has a price tag of 1.1 million US dollars. This advanced therapy, along with five of the other seven EU-authorized advanced therapies, has yet to achieve national reimbursement in the EU. Uniqure, the developers of Glybera, the world’s first approved gene therapy, recently announced that it will not request renewal of its market authorization with the EMA. Therefore, that leaves ChondroCelect, a tissue engineered therapy based on autologous cells as the only advanced therapy to have achieved national reimbursement in the EU but only in three countries, namely Spain, Belgium, and the Netherlands. This may, however, be due to its more modest price tag of 20,000 US dollars in comparison to Glybera and the 93,000-US-dollar priced Provenge, another autologous-based treatment, and Strimvelis, an *ex vivo* hematopoeitic stem cell therapy costing 594,000 US dollars (Abou-El-Enein et al., 2016a). With these complexities, similarly with manufacturing, the developer needs to begin to address the issue of reimbursement at the very beginning of the translational process.

### Availability of Expertise Not Related to Basic Research in Academic Institutions

Often overlooked is the fact that the development of a novel drug is a strictly regulated affair requiring several expertise areas which are rarely present in academic institutions. In the field of the ATMP, a few GMP manufacturing centers have been developed but their maintenance is hugely expensive and rarely supported by sufficient public funding. Very few institutions have access to the financial, commercial, and operational resources necessary for successful market entry. Most of them deal with this issue by relying on industry to pick up the IP and translate it to the market. However, they often fail due to weaknesses in the translational research strategy required to bring them to a value point sufficiently mature to be of interest for industry. These weaknesses are numerous, but include elements such as poor resource planning, IP protection, regulatory planning, often brought about by the absence of a rigorous reverse planning approach to the development plan (Driscoll et al., 2017).

In light of the above, publicly funded translational medicine proposals should also address value assessments which offer a real and immediate consideration for access to major markets and are assessed as part of the clinical translation process. There are multiple components to an assessment, such as the example of the Value-Engineered Translation (VET) Framework, that develops biotherapeutics that align with healthcare system needs and formally integrates available analytics. These components are already being adopted by supportive organizations such as the Cell Therapy Catapult, CCRM, technology transfer offices, patent offices, and HTA agencies (Bubela et al., 2015). The VET Framework could provide an analysis of any unmet need for a candidate ATMP for a specific disease to support a price consistent with an acceptable return on the investment in clinical translation. This analytical approach depends on pools of data, communications by networks and groups operating in advanced therapies, journal articles, clinical trial registries, regulatory approvals, and commercial databases. This information is then filtered based on the innovativeness of the new technology relative to current practice, expected time to availability and potential for clinical, patient, cost, and staffing impacts.

### Role of the European Agencies and the Research Infrastructures

**Figure 1:**
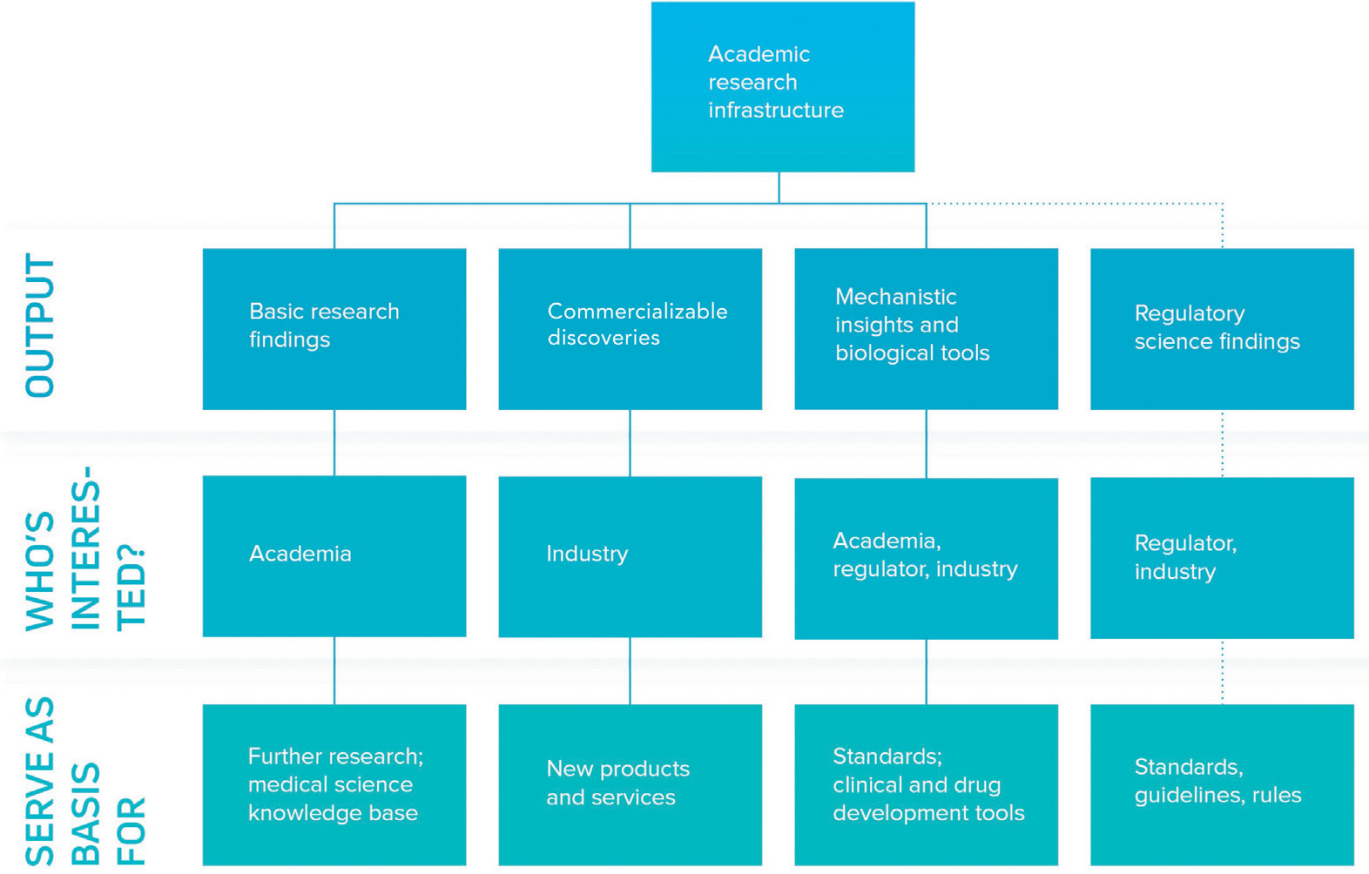
Academia in the knowledge and innovation cascade. Academia plays an important role in the generation of novelty in the advanced therapy medicinal product (ATMP) space. The first three strands of the innovation cascade are well established, with academic publication mediating delivery of basic research findings and novel biology and tools, with intellectual property (most commonly in the form of patents or industrial secrets) serving as a vehicle for commercializable discoveries. The fourth strand depicting a regulatory science framework is poorly developed in Europe but a pressing need in ATMP development and would require structural dialogue between regulator, academia and their funders, and industry. Research infrastructures have a role to play in defragmenting the academic field, sourcing independent labs, and acting as a trusted third party in the interplay between regulator and industry.

Given the scientific novelty of most ATMP approaches, there is a dearth of standards to guide the development and manufacturing process. As a consequence, of enormous importance is the need for optimal dialog between regulator, independent research laboratories and ATMP developer. Such a tripartite initiative is required to allow the regulator to pose scientific questions that come up during the normal authorization process, to be answered by lab work conducted in facilities whose owners have no conflict of interest. The results can not only inform the specific case from which the question arose but also be used as the basis for future guidelines or rules. Any such effort that can reduce uncertainty by generating clarity on the levels of evidence required to pass regulatory muster will be of great value to the system. Naturally, it is essential that any such framework would allow such knowledge generation and exchange to happen quickly, so that innovation is not stifled. Figure 1 shows a knowledge and innovation cascade comprising academia, industry and regulator; where today the fourth stream—as described above—is yet to be put into practice in Europe.

One recent important initiative to establishing greater cooperation and knowledge sharing between the regulator and academic stakeholders is the EMA *framework of collaboration with academic stakeholders*. A workshop was held in 2016 to discuss and collect input for this new framework of collaboration (Ussi and Migliaccio, 2016). One interesting concept discussed was that of creating a framework whereby regulatory science questions emerging from the review process could be captured and prioritized in a research agenda and subsequently executed within independent labs, through financing from third parties such as the European Commission.

We define regulatory science here as that of validating the findings and analytical tools required for confident efficacy and safety evaluation. Such a field is mostly independent from a specific product and needs specific expertise linked to the validation process, performed in a quality assurance system which is strictly regulated by national and European laws. Sadly, the field of regulatory science is presently drastically underfunded by public sources, making the EMA initiative all the timelier. Although there is interest behind this strategy, momentum and the means to finance such an approach have not yet been evoked. The development of research partnerships supported by funding mechanisms such as the Innovative Medicines Initiative partially cover these needs, as the main focus is on increasing precompetitive innovation and basic research questions rather than specific questions such as the ones discussed here in optimizing ATMP production. There is a clear funding gap for such academia-led confirmatory studies, which is acting as a drag on the system as the lack of robust validation for discovery work leads to wasted efforts by both academia and industry. Therefore, funding designated for addressing the research questions set by the regulatory agencies would be of great value.

The concept of working closely with academics to address these gaps in ATMP manufacturing will require multi-national commitment from academia and regulators. To initiate such a collaboration around this topic, expert meetings would first be most appropriate to get more diverse feedback on the whole manufacturing process. Presently in the UK for example (Advanced Therapies Manufacturing Taskforce, 2016), there is already activity to this end, however this cannot be done on national scale alone as it pertains to development of a product that would be distributed on an international scale. Therefore, it is necessary to address, for example, how GMP would work in a distributed model across national boundaries, and thus across multiple regulatory jurisdictions. The existing European-wide Research Infrastructures such as EATRIS ERIC, which is focused on translational development of novel therapeutics and diagnostics and supported and owned by the member states, could play a fundamental role in answering regulatory questions about the development of an ATMP on an international scale. A consortium consisting of key opinion leaders in all facets of ATMP development, in addition to networks such as the learned societies including the International Society of Cell Therapies and the Parenteral Drug Association for instance, could play an integral part in facilitating such initiatives, together with the EMA and the Innovation Offices recently initiated by most European national competent authorities. Such organizations are necessary as they can provide:

- Critical mass by representing many or most players in a given field.
- Resources for the coordination of activities, for efficient operations.
- Fast access to a broad range of expertise and facilities, to enable quick execution of research.
- Trusted third party status to prevent potential conflict of interest situations, particularly of the European Research Infrastructure Consortium legal entity utilized by infrastructures such as EATRIS.

EU funded projects directed toward utilization of such a framework would allow regulators to generate data about the some of the major issues of the ATMP development process, such as distribution or scale-up. Presently in the field there are no experience-based guidelines, just large blocks of automation, such as in hematology for cell separation. Research infrastructures could, however, compare automation across groups, addressing the complicated area of decentralized manufacturing. Such projects would aim to ascertain the regulatory requirements from one center to the next to ensure comparability and quality demanded of batch release. Several companies are actively involved in “GMP in a box” as a possible solution which gives rise to additional questions, such as the type of warranty that would be given for such machines against changes in viability.

Comparability is, however, very complex in regulatory terms, and generally taken to mean “of equal risk benefit.” As a result, research projects dedicated to find the necessary parameters to process this specific product would be required to inform on the quality critical parameters and the drivers of variability therein. Such projects should aim to deliver robust data that report consistency and limits of standardization, which includes production and release parameters. It is worth reiterating that it is imperative for the regulator and other ATMP manufacturing stakeholders to always be in dialog in this process, to continuously ensure that product, manufacturing model, and regulatory expectations remain aligned.

These issues are clearly not limited to the European market or scientific environment but are in common with all the advanced economies (Ecorys Nederland and University Utrecht, 2016; Phacilitate, 2017). In the USA, the Advanced Therapy products are a class of Biologicals with a regulatory approach based on a risk assessment more akin to the Cell and Tissue framework in Europe. In Japan, the restructuring of the Regulatory Agencies was coincidental with strong government support for the application to medicine of iPS and the creation of a network of universities dedicated to translational medicine. However, both areas as well as Australia and Canada, have a similar regulatory framework in place for Advanced Therapies to that of Europe, with no substantial differences (Ecorys Nederland and University Utrecht, 2016; Papadaki, 2017).

In South East Asia, countries such as South Korea and Taiwan are moving in the same direction, asking progressively more in terms of Quality and Efficacy to the clinician proposing new treatments with Advanced Therapies.

To address the complexity of reimbursement for advanced therapies, there is a need for constant dialog among all the actors. This includes clinician researchers, patients, regulators, HTA bodies, and payers. Moreover, the mapping of existing resources such as registries, Hospital Exemptions, and compassionate use records and the use of real world evidence of clinical utility is fundamental to assess the long-term efficacy required. As with manufacturing, an ATMP adoption pathway strategy in establishing viable funding and stakeholder engagement routes to address the issue of reimbursement is required. Innovative partnerships with commensurate access to resources are required to formulate strategies to ease this roadblock to the clinical utilization of these therapies (Advanced Therapies Manufacturing Taskforce, 2016; Papadaki, 2017; Phacilitate, 2017).

Funded projects on answering issues in the manufacturing space could be coupled to deliverables on reimbursement questions, again demanding a concerted effort of all stakeholders. This collective approach requires the input of multiple group types across multiple countries and sufficient funding to establish a framework for future reimbursement strategies (Advanced Therapies Manufacturing Taskforce, 2016). It is here again that pan European research infrastructures can add most value to this type of data collection from multiple sources, as multiple groups operating on ATMPs and clinical trial registries across Europe can be collated more efficiently, leading to the establishment of a VET-like framework for reimbursement. Similarly, to the gaps around ATMP manufacturing, the regulatory authorities would also be pivotal to early development of a pan European framework for reimbursement. Hence, the collective effort of developer, funder, regulator, and research infrastructure is key for the development of such an initiative. The collaborative feedback of these funded initiatives and the resulting deliverables from these funded projects will hopefully facilitate the development of novel regulatory and reimbursement models to support the development of the advanced therapies industry, ensuring its transition from a cottage type to a commercial one.

Only with adequate commitment and funding mechanisms involving the correct stakeholders to develop these funding calls will we begin to find solutions to these now well-documented gaps in the commercialization of these necessary advanced therapies. This article supports the need for an innovation group which involves the industry, funder, regulator, academic research infrastructures to address the systemic challenges and their project-specific manifestations, where only a concerted approach will yield the necessary insights and breakthroughs.

## Author Contributions

Each author contributed equally.

## Conflict of Interest Statement

The authors declare that the research was conducted in the absence of any commercial or financial relationships that could be construed as a potential conflict of interest. The reviewer SM and handling editor declared their shared affiliation.

## References

[B1] Abou-El-Enein M. Elsanhoury A. Reinke P. (2016a). Overcoming challenges facing advanced therapies in the EU market. Cell Stem Cell 19, 293–297. 10.1016/j.stem.2016.08.012 27588746

[B2] Abou-El-Enein M. Bauer G. Medcalf N. Volk H. D. Reinke P. (2016b). Putting a price tag on novel autologous cellular therapies. Cytotherapy 18, 1056–1061. 10.1016/j.jcyt.2016.05.005 27288308

[B3] Advanced Therapies Manufacturing Taskforce. (2016). Advanced-Therapies-Manufacturing-Taskforce-Report. Available at: http://www.abpi.org.uk/our-work/mmip/Documents/Advanced-Therapies-Manufacturing-Taskforce-report.pdf

[B4] Bubela T. McCabe C. Archibald P. Atkins H. Bradshaw S. E. Kefalas P. (2015). Bringing regenerative medicines to the clinic: the future for regulation and reimbursement. Regen. Med. 10, 897–911. 10.2217/rme.15.51 26565607

[B5] Driscoll D. Farnia S. Kefalas P. Maziarz R. T. (2017). Concise review: the high cost of high tech medicine: planning ahead for market access. Stem Cells Transl. Med. 6, 1723–1729. 10.1002/sctm.16-0487 28749065 PMC5689744

[B6] Ecorys Nederland and University Utrecht. (2016). Study on the Regulation of Advanced Therapies in Selected Jurisdiction. Brussels: RfS Chafea, European Union, 0–327. Final Report. ISBN: 978-92-9200-731-7.

[B7] FDA. (2017). Approved Products. Available at: https://www.fda.gov/BiologicsBloodVaccines/CellularGeneTherapyProducts/ApprovedProducts/default.htm

[B8] Fellmann C. Gowen B. G. Lin P. C. Doudna J. A. Corn J. E. (2017). Cornerstones of CRISPR–Cas in drug discovery and therapy. Nat. Rev. Drug Discov. 16, 89–100. 10.1038/nrd.2016.238 28008168 PMC5459481

[B9] Heathman T. R. Nienow A. W. McCall M. J. Coopman K. Kara B. Hewitt C. J. (2015). The translation of cell-based therapies: clinical landscape and manufacturing challenges. Regen. Med. 10, 49–64. 10.2217/rme.14.73 25562352

[B10] Hourd P. Ginty P. Chandra A. Williams D. J. (2014). Manufacturing models permitting roll out/scale out of clinically led autologous cell therapies: regulatory and scientific challenges for comparability. Cytotherapy 16, 1033–1047. 10.1016/j.jcyt.2014.03.005 24856894

[B11] IMI. (2016). Outcomes of the IMI Consultation on Advanced Therapies. Brussels: IMI.

[B12] Kamenova K. Caulfield T. (2015). Stem cell hype: media portrayal of therapy translation. Sci. Transl. Med. 7, 278s4. 10.1126/scitranslmed.3010496 25761887

[B13] Papadaki M. (2017). Adaptation through collaboration: developing novel platforms to advance the delivery of advanced therapies to patients. Front Med 4:56. 10.3389/fmed.2017.00056 28611985 PMC5447030

[B14] PEI. (2016). Advanced Therapy Medicinal Products (ATMP). Available at: http://www.pei.de/EN/medicinal-products/advanced-therapy-medicinal-products-atmp/advanced-therapy-medicinal-products-atmp-node.html

[B15] Phacilitate. (2017). Advanced Therapies Investment Report 2017. Available at: http://ccrm.ca/sites/default/files/pdfs/Investment_for_Advanced_Therapies_Report.pdf

[B16] Sensebé L. Gadelorge M. Fleury-Cappellesso S. (2013). Production of mesenchymal stromal/stem cells according to good manufacturing practices: a review. Stem Cell Res Ther. 4, 66. 10.1186/scrt217 23751270 PMC3707032

[B17] Thomson J. A. Itskovitz-Eldor J. Shapiro S. S. Waknitz M. A. Swiergiel J. J. Marshall V. S. (1998). Embryonic stem cell lines derived from human blastocysts. Science 282, 1145–1147. 10.1126/science.282.5391.1145 9804556

[B18] Trounson A. McDonald C. (2015). Stem cell therapies in clinical trials: progress and challenges. Cell Stem Cell 17, 11–22. 10.1016/j.stem.2015.06.007 26140604

[B19] Ussi A. Migliaccio G. (2016). The EMA framework of collaboration with academic stakeholders. Mol. Ther. 24, 1883–1884. 10.1038/mt.2016.187 27916997 PMC5154483

[B20] Wainwright S. P. Williams C. Michael M. Farsides B. Cribb A. (2006). Ethical boundary-work in the embryonic stem cell laboratory. Sociol. Health Illn. 28, 732–748. 10.1111/j.1467-9566.2006.00539.x 17184415

